# Control of antigen receptor gene assembly by cis-element mediated looping

**DOI:** 10.1186/1756-8935-6-S1-P51

**Published:** 2013-03-18

**Authors:** Kinjal Majumder, Olivia Koues, Suhasni Gopalakrishnan, Oleg Osipovich, Yue Huang, Eugene Oltz

**Affiliations:** 1 Department of Pathology and Immunology, Washington University School of Medicine, Saint Louis, Missouri, 63110, USA

## 

The adaptive immune system endows mammals with a sophisticated mechanism to survive in a world filled with pathogens. This dynamic defense is generated by V(D)J recombination, which enables lymphocytes to uniquely interact with an enormous range of epitopes on foreign antigens. V(D)J recombination is a set of sequentially controlled DNA cleavage and repair events, which brings variable (V), diversity (D) and joining (J) gene segments together to form functional antigen receptor genes. However, the recombination process must be stringently regulated to prevent formation of chromosomal translocations, which can lead to tumors. The process of V(D)J recombination is regulated at the level of tissue, stage and allele specificity by genetic and epigenetic mechanisms. Our lab has characterized several genetic elements that regulate chromatin accessibility and recombination at the T cell receptor beta (*Tcrb*) locus. These include transcriptional promoters and enhancers, which interact with each other in conformational space to form a promoter-enhancer holocomplex. The holocomplex subsequently recruits the SWI/SNF chromatin remodeling complex, leading to locus activation. However, the factors that mediate conformation and epigenetic changes required for efficient *Tcrb* gene assembly are unknown. We hypothesized that active transcription may stabilize the holocomplex. In order to test this, we chemically induced promoter-proximal-pausing in proT cells. We discovered that in the absence of elongating RNA polymerase II, the promoter-enhancer holocomplex is disrupted without any robust changes in transcription factor binding, structural protein recruitment or histone acetylation across the locus. Ongoing work seeks to decipher whether the transcriptional machinery is required at all for holocomplex stability. Meanwhile, in order to dissect the spatial and epigenetic mechanisms that lead to *Tcrb* activation, our lab has generated mutated versions of *Tcrb* cis-elements in a proT cell line model using Zinc Finger Nucleases. Preliminary studies on these mutants have shown that the holocomplex formation requires the enhancer (or enhancer bound elements) and also CTCF sites flanking the locus. Ongoing studies seek to identify which CTCF sites are necessary and sufficient for holocomplex stability. Using these and additional mutants, we are testing the role played by cis-elements in maintaining the chromatin and looping of *Tcrb*, which are essential for recombination.

